# Comparative analysis of microbial communities in different growth stages of *Dermacentor nuttalli*

**DOI:** 10.3389/fvets.2022.1021426

**Published:** 2022-10-14

**Authors:** Li Zhao, Yi-Min Ma, Bo Yang, Wen-Xiong Han, Wei-Hong Zhao, Hai-Liang Chai, Zhan-Sheng Zhang, Yong-Jie Zhan, Li-Feng Wang, Yu Xing, Lu-Fei Yu, Jin-Ling Wang, Yu-Lin Ding, Yong-Hong Liu

**Affiliations:** ^1^College of Veterinary Medicine, Inner Mongolia Agricultural University, Hohhot, China; ^2^Key Laboratory of Clinical Diagnosis and Treatment Technology in Animal Disease, Ministry of Agriculture and Rural Affairs, Hohhot, China; ^3^Animal Disease Control Center of Ordos, Ordos City, China; ^4^Inner Mongolia Saikexing Reproductive Biotechnology (Group) Co., Ltd., Hohhot, China; ^5^Shanghai Origingene Bio-pharm Technology Co. Ltd., Shanghai, China

**Keywords:** tick, *Dermacentor nuttalli*, growth stage, microbial population, high-throughput sequencing

## Abstract

Ticks were identified as arthropods that are pathogenic vectors. *Dermacentor nuttalli* is one of the dominant tick species in Inner Mongolia, and it carries and transmits a wide range of pathogenic microorganisms. However, at present, only the detection of *D. nuttalli* adult ticks and *D. nuttalli* different developmental stages carrying one specific pathogen, or the next-generation sequencing of *D. nuttalli* adult ticks were available. In this study, we investigated the microbial community structures of *D. nuttalli* in different growth stages under laboratory artificial feeding conditions. Total DNA was extracted from seven growth stages (female adult ticks, eggs, larval ticks, engorged larval ticks, nymphal ticks, engorged nymphal ticks, and second-generation adult ticks) obtained from laboratory artificial feeding of engorged *D. nuttalli* female ticks in Inner Mongolia. Then, the 16S rDNA V3–V4 hypervariable region was amplified to construct an Illumina PE250 library. Finally, 16S rRNA sequencing was performed on Illumina Novaseq 6000 platform. The sequencing data were analyzed using molecular biology software and platforms. The Illumina PE250 sequencing results showed that the egg stage had the highest diversity and number of species (28.74%, 98/341), while the engorged nymph stage had the lowest diversity and number of species (9.72%, 21/216). A total of 387 genera of 22 phyla were annotated in *D. nuttalli*, with 9 phyla and 57 genera found throughout all 7 growth stages. The dominant phylum was Proteobacteria; the dominant genera were *Arsenophonus* and *Rickettsia*; and the genera with the highest relative abundance in the 7 growth stages were *Pseudomonas, Paenalcaligenes, Arsenophonus, Arsenophonus, Pseudomonas, Arsenophonus*, and *Rickettsia*, respectively. Among the 23 exact species annotated, *Brucella melitensis* exhibits pathogeny that poses a serious threat to humans and animals. In this study, the microbial community composition at different growth stages of *D. nuttalli* was comprehensively analyzed for the first time.

## Introduction

Ticks are bloodsucking ectoparasites found on mammalian, avian, reptilian, and amphibian body surfaces (1). Ticks are classified into 4 families (*Ixodidae, Argasidae, Nuttalliellidae*, and *Deinocrotonidae*), with 949 species of ticks worldwide, including 731 species of *Ixodidae* (2). China recorded 2 families of 124 tick species, of which 113 were hard-tick species. Ticks are the most common in Central China, South China, and Inner Mongolia-Xinjiang, with 56, 54, and 46 hard-tick species, respectively (3). Ticks from the family *Ixodidae* are the most common and harmful, with four developmental stages: egg, larva, nymph, and adult, with the larva, nymph, and adult stages requiring host blood to complete their development (4). Ticks can directly harm the host by feeding on their blood; Additionally, they can carry and transmit pathogenic microorganisms such as bacteria, viruses, protozoan parasites, and spirochetes (5–8). They are the world's second largest group of vectors, after mosquitoes (9, 10).

*Dermacentor nuttalli* belongs to the family Ixodidae and genus *Dermacentor*, and it is one of the dominant tick species in the Inner Mongolia region (11). In addition to the Inner Mongolia region, *D. nuttalli* individuals carrying multiple microorganisms have been identified in the Xinjiang, Yunnan, Gansu, Shaanxi, Shanxi, Heilongjiang, and Jilin Province, etc. Moreover, *D. nuttalli* individuals carrying microorganisms have been found in the neighboring countries of Mongolia and Russia (Table 1). The above studies are sufficient to prove that *D. nut*talli has a strong ability to carry and transmit pathogenic microorganisms. Different species of ticks prefer distinct biotopes or environments, which determines the geographic distribution of ticks (10). This is coupled with the fact that different tick species carry and transmit different pathogens (12). Hence, a comprehensive analysis of microbial communities transmitted by *D. nuttalli* in Inner Mongolia is important for assessing the risk of local tick-borne diseases.

**Table 1:** Microorganisms carried by *Dermacentor nuttalli* in different geographical regions.

**Region**	**Microorganism**	**References**
Inner Mongolia	*Anaplasma* sp. Mongolia	(41)
	*Babesia venatorum*	(41)
	*Borrelia miyamotoi*	(21)
	*Brucella* spp.	(34)
	*Coxiella-*like endosymbiont	(41)
	*Rickettsia aeschlimannii*	(18)
	*Rickettsia heilongjiangensis*	(10)
	*Rickettsia raoultii*	(18, 41)
	*Rickettsia tarasevichiae*	(41)
Xinjiang, Yunnan, Gansu, Shaanxi, Shanxi, Heilongjiang, and Jilin Province, etc.	*Acinetobacter pittii*	(44)
	*Anaplasma ovis*	(22, 30)
	*Arthrobacter citreus*	(44)
	*Arthrobacter gandavensis*	(44)
	*Babesia caballi*	(10, 22)
	*Babesia occultans*	(30)
	*Bacillus cereus*	(44)
	*Bacillus infantis*	(44)
	*Bacillus pumilus*	(44)
	*Bacillus simplex*	(44)
	*Bacillus thuringiensis*	(44)
	*Borrelia afzelii*	(28)
	*Borrelia bavariensis*	(28)
	*Borrelia bissettii*	(28)
	*Borrelia garinii*	(28)
	*Brevundimonas vesicularis*	(44)
	*Brucella* sp.	(22)
	*Candida lipolytica*	(44)
	Candidatus *Rickettsia tibetani*	(27)
	*Cellulosimicrobium cellulans*	(44)
	*Corynebacterium glutamicum*	(44)
	*Corynebacterium mucifaciens*	(44)
	*Coxiella burnetii*	(26)
	*Delftia acidovorans*	(44)
	*Ewingella americana*	(44)
	*Exiguobacterium aurantiacum*	(44)
	*Janibacter hoylei*	(44)
	*Microbacterium liquefaciens*	(44)
	*Microbacterium oxydans*	(44)
	*Microbacterium paraoxydans*	(44)
	*Microbacterium phyllosphaerae*	(44)
	*Microbacterium testaceum*	(44)
	*Micrococcus luteus*	(44)
	*Paenibacillus amylolyticus*	(44)
	*Pantoea agglomerans*	(44)
	*Pseudoclavibacter helvolus*	(44)
	*Pseudomonas chlororaphis*	(44)
	*Pseudomonas extremorientalis*	(44)
	*Pseudomonas fluorescens*	(44)
	*Pseudomonas frederiksbergensis*	(44)
	*Pseudomonas grimontii*	(44)
	*Pseudomonas kilonensis*	(44)
	*Pseudomonas koreensis*	(44)
	*Pseudomonas libanensis*	(44)
	*Pseudomonas marginalis*	(44)
	*Pseudomonas orientalis*	(44)
	*Pseudomonas putida*	(44)
	*Pseudomonas synxantha*	(44)
	*Pseudoxanthomonas kaohsiungensis*	(44)
	*Pseudochrobactrum saccharolyticum*	(44)
	*Rickettsia raoultii*	(16, 22–25, 27, 38, 39)
	*Rickettsia sibirica*	(23)
	*Rickettsia sibirica* subsp. *Sibirica*	(27, 39)
	*Rickettsia slovaca*	(23)
	*Rickettsia* sp. 10CYF	(27)
	*Rhodococcus erythropolis*	(44)
	*Sanguibacter inulinus*	(44)
	*Serratia marcescens*	(44)
	*Staphylococcus epidermidis*	(44)
	*Staphylococcus nepalensis*	(44)
	*Staphylococcus warneri*	(44)
	*Stenotrophomonas rhizophila*	(44)
	*Theileria ovis*	(22)
	Blacklegged tick phlebovirus	(43)
	Crimean-Congo Hemorrhagic fever virus	(96)
	deer tick Mononegavirales-like virus	(43)
	Guertu virus	(33)
	Jingmen tick virus	(43)
	lymphocytic choriomeningitis virus	(29)
	South Bay virus	(43)
	Tacheng tick virus 1	(31)
Mongolia and Russia	*Anaplasma ovis*	(17)
	*Anaplasma phagocytophilum*	(32)
	*Anaplasma* sp. Mongolia	(37)
	*Anaplasma* spp.	(20)
	*Babesia caballi*	(32, 37)
	*Borrelia afzelii*	(37)
	*Candidatus Midichloria* sp.	(37)
	*Candidatus Neoehrlichia mikurensis*	(37)
	Crimean-Congo Hemorrhagic fever virus	(15)
	*Theileria equi*	(32, 37)
	*Theileria orientalis*	(37)
	tick-borne encephalitis virus	(35)
	*Rickettsia raoultii*	(19, 37, 40, 66)
	*Rickettsia sibirica*	(19)
	*Rickettsia sibirica* subsp. *Sibirica*	(66)
	Wad Medani virus in Russia	(42)
	Yanggou tick virus	(36)

High-throughput sequencing technology is quick and efficient for analyzing tick microbial communities, thereby eliminating the need for bacterial culture techniques and allowing the identification of non-culturable bacteria and unknown pathogens (13). The study of 16S rDNA regions can be used to identify species and as indicators in microbial systematics, classification, and identification. The V3–V4 hypervariable regions are the most accurate for identifying organisms at the genus level (14). Previous studies have mainly focused on detecting *D. nuttalli* adult ticks (15–33) and *D. nuttalli* different developmental stages (34–40) carrying one specific pathogen or on next-generation sequencing of *D. nuttalli* adult ticks (41–44). The microbial community structure and diversity of *D. nuttalli* at different developmental stages have not been comprehensively compared. In this study, we used different growth stages of *D. nuttalli* as part of the study objectives under laboratory artificial feeding conditions and sequenced the V3–V4 hypervariable regions based on the 16S rDNA region using the Illumina Novaseq 6000 platform to determine the microbial community structure and diversity of *D. nuttalli* at various growth stages and to provide a scientific basis for risk assessment and prevention of *D. nuttalli* and its tick-borne diseases.

## Materials and methods

### Study areas and sample collection

**Figure 1:**
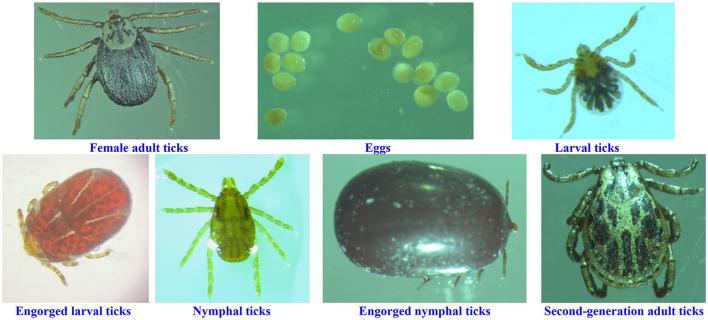
Ticks at different growth stages in this study.

Ticks used in this study were from a colony of *D. nuttalli* maintained at the Tick Research Laboratory, Inner Mongolia Agricultural University, College of Veterinary Medicine, Hohhot, using mice as hosts for all growth stages. This colony originated from field collections of ticks from the same sheep in New Barag Left Banner, Hulunbeier, Inner Mongolia (619 m above sea level; 48°48′N, 118°24′E), who had no other obvious abnormalities except emaciation. Ticks were manually pulled from the sheep using forceps and placed directly into a 50 mL centrifuge tube, after which the nozzle was plugged close with damp cotton and transported to the laboratory. Engorged adult female *D. nuttalli* ticks were identified in the laboratory using morphological and molecular biological methods. The remaining six growth stages (egg, larval ticks, engorged larval ticks, nymphal ticks, engorged nymphal ticks, and second-generation adult ticks) were obtained from the colony in the laboratory (Figure 1), and three replicates of each stage were set up (each sample was mixed), yielding a total of 21 samples stored at −80°C.

### DNA extraction

Each sample was washed three times in 70% ethanol and sterile deionized water, followed by extraction using the TaKaRa MiniBEST Universal Genomic DNA extraction kit in the biosafety cabinet. The 5.0 (Takara, Beijing, China, Code No. 9765) kit was used to extract total DNA from samples based on the manufacturer's instructions, and the DNA was stored temporarily at −20°C.

### Polymerase chain reaction amplification and high-throughput sequencing

The 16S rDNA V3–V4 region was amplified using the *TransStart*^®^ *FastPfu* DNA polymerase (TransStart, Beijing, China, Code No. AP221-02) kit, using sample DNA as a template and specific barcode primers. The primer was 338F: 5'-ACTCCTACGGGAGGCAGCA-3′, 806R: 5′-GGACTACHVGGGTWTCTAAT-3′. The annealing temperature was 55°C. The PCR products were recovered by gel extraction using the AXYPREP DNA gel extraction kit (AXYGEN, Suzhou, China, Code No. AP-GX-50G), which were tested for conformance using 2% agarose gel electrophoresis, and then the VAHTS^®^ ssDNA Library Prep kit (Illumina, San Diago, USA, Code No. ND6201) was used to construct the Illumina PE250 library, followed by high-throughput sequencing of 16S rDNA on the Illumina Novaseq 6000 platform (San Diego, CA, USA).

### Data analysis

For Illumina PE250 sequencing sequences, we first obtained valid sequences for all samples based on the barcode. Then, paired-end reads from the original DNA fragments were merged using FLASH (version 1.2.11). Sequence analysis was performed using the UPARSE software package (version 7.0.1090 http://drive5.com/uparse/) (45). Based on sequences with ≥97% similarity, all sequences were categorized and operational taxonomic units (OTUs) were generated. The RDP Classifier version 2.2 (http://sourceforge.net/projects/rdp-classifier/) (46) bayesian algorithm was used to perform taxonomic analysis of OTU representative sequences from domain to species. For single-sample diversity analysis (alpha diversity), we calculated the number of unique OTUs in each sample, the Chao index of community richness [the Chao1 estimator (http://www.mothur.org/wiki/Chao)], the Shannon index for community diversity (http://www.mothur.org/wiki/Shannon), Simpson index (http://www.mothur.org/wiki/Simpson), and the sequencing depth coverage [the Good's coverage (http://www.mothur.org/wiki/Coverage)]. Species accumulation curves were combined to determine whether the sample size was sufficient to assess and predict species richness (47). The number of common and unique OTUs in multiple samples was counted using the R language and displayed in Venn diagrams (48). The composition of microbial communities was presented using histograms based on OTU data using the R language (49).

Sequencing and sequence analysis were performed in collaboration with Origin-gene biology Co., Ltd. (Shanghai, China).

## Results

### General statistics

The sequencing results of all samples were corrected, and chimeras were removed to obtain 3,437,890 optimized sequences, including 499,595 from female adult ticks; 518,688 from eggs; 534,255 from larval ticks; 492,368 from engorged larval ticks; 519,116 from nymphal ticks; 472,277 from engorged nymphal ticks; and 401,591 from second-generation adult ticks. The base number of these optimized data was 1,452,643,023 bp. The average sequence length of these optimized data was 422.54 bp, and 99.92% of the sequences were mapped to the 401–440 bp regions. There were 3,305,190 high-quality, effective, where clean reads were generated for the analysis using OTU selection and taxonomic assignments (Table 2).

**Table 2:** Statistics of sequencing result.

**Parameter**	**Female adult**	**Eggs**	**Larval**	**Engorged**	**Nymphal**	**Engorged**	**Second-generation**	**Total**
	**ticks**		**ticks**	**larval ticks**	**ticks**	**nymphal ticks**	**adult ticks**	
Sequences	499,595	518,688	534,255	492,368	519,116	472,277	401,591	3,437,890
Bases (bp)	210,687,653	220,507,211	226,008,992	206,612,314	219,777,058	200,604,061	168,445,734	1,452,643,023
Average length (bp)	421.72	425.12	423.04	419.63	423.37	424.76	419.45	422.54
Clean reads	460,571	490,731	527,966	485,505	488,530	467,464	384,423	3,305,190

### Alpha-diversity analysis

**Figure 2:**
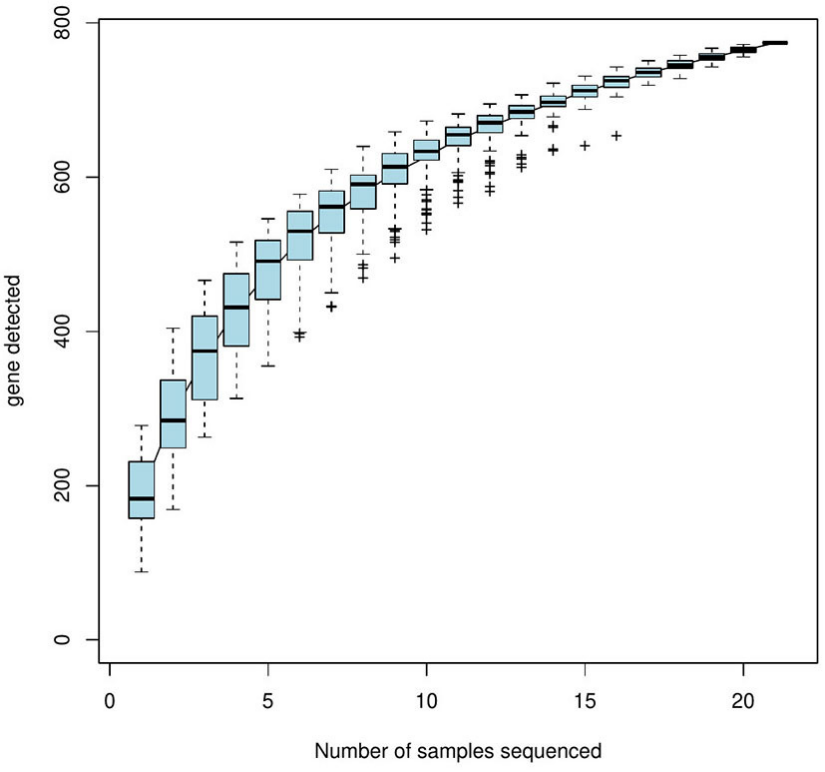
Species accumulation curves (horizontal coordinate, sample size, vertical coordinate, and number of OTUs after sampling).

The alpha-diversity indices (Shannon, Simpson, Chao, ACE, and Good's coverage) were calculated based on a 97% similarity level, which showed that the Shannon index values were eggs > nymphal ticks > larval ticks > second-generation adult ticks > female adult ticks > engorged larval ticks > engorged nymphal ticks; the Simpson index values were eggs < nymphal ticks < larval ticks < second-generation adult ticks < female adult ticks < engorged larval ticks < engorged nymphal ticks; and the Chao and ACE indices were eggs > larval ticks > nymphal ticks > engorged larval ticks > female adult ticks > second-generation adult ticks > engorged nymphal ticks. The Good's coverage of all samples exceeded 99.9% (Table 3). The species accumulation curves (Figure 2) showed that as sample size increased, the curves first increased sharply and then plateaued.

**Table 3:** Community richness and alpha-diversity indices of the samples.

**Sample**	**0.97**			
	**Good's**	**Shannon**	**Simpson**	**Chao**	**ACE**
	**coverage (%)**	**index**	**index**	**index**	**index**
Female adult ticks	99.97	1.51	0.40	200	214
Eggs	99.97	3.20	0.08	322	328
Larval ticks	99.97	2.17	0.26	274	272
Engorged larval ticks	99.97	0.98	0.47	213	218
Nymphal ticks	99.97	2.40	0.20	270	263
Engorged nymphal ticks	99.97	0.33	0.86	155	164
Second-generation adult ticks	99.97	1.94	0.28	184	192

### OTU cluster analysis

**Figure 3:**
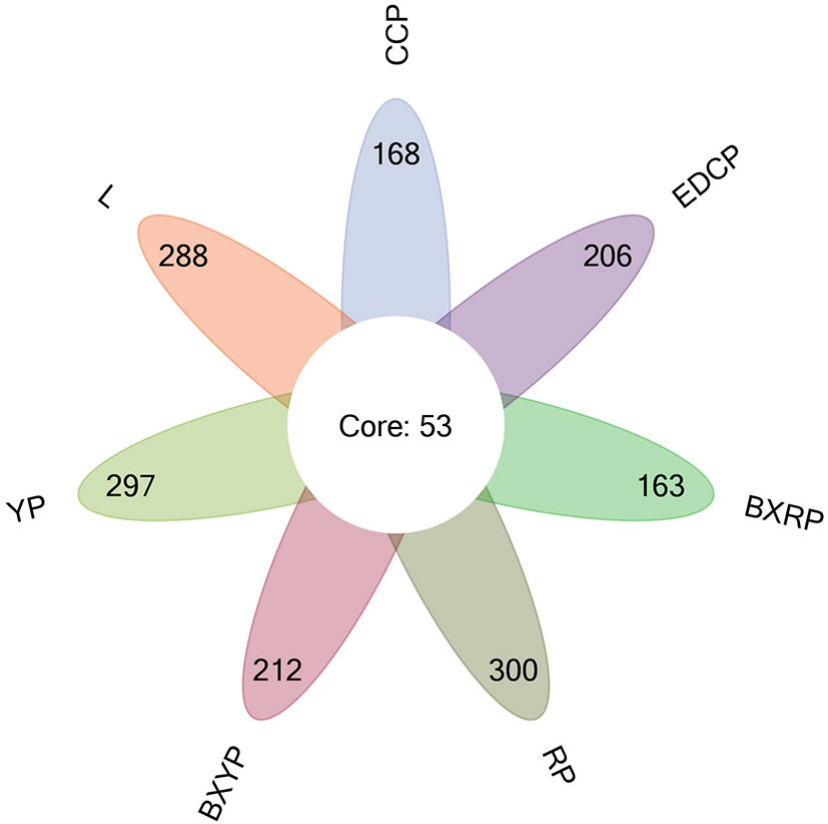
Venn diagram of seven samples based on OTUs (CCP, female adult ticks; L, eggs, YP, larval ticks; BXYP, engorged larval ticks; RP, nymphal ticks; BXRP, engorged nymphal ticks; EDCP, second-generation adult ticks).

The Venn diagram (Figure 3) shows that 221, 341, 350, 265, 353, 216, and 259 OTUs were obtained for female adult ticks, eggs, larval ticks, engorged larval ticks, nymphal ticks, engorged nymphal ticks, and second-generation adult ticks, respectively. Of these, 53 OTUs showed high similarity in the 7 growth stage samples of *D. nuttalli*, and the unique OTUs for the 7 growth stage samples were 25 (11.31%, 25/221), 98 (28.74%, 98/341), 45 (12.86%, 45/ 350), 33 (12.45%, 33/265), 49 (13.88%, 49/353), 21 (9.72%, 21/216), and 28 (10.81%, 28/259), respectively. In all samples, most tags were classified and >87.07% of tags were assigned to the genus level. Only a small percentage of tags could be accurately classified at the species level.

### Microbial population

#### Microbial community composition at the phylum level

**Figure 4:**
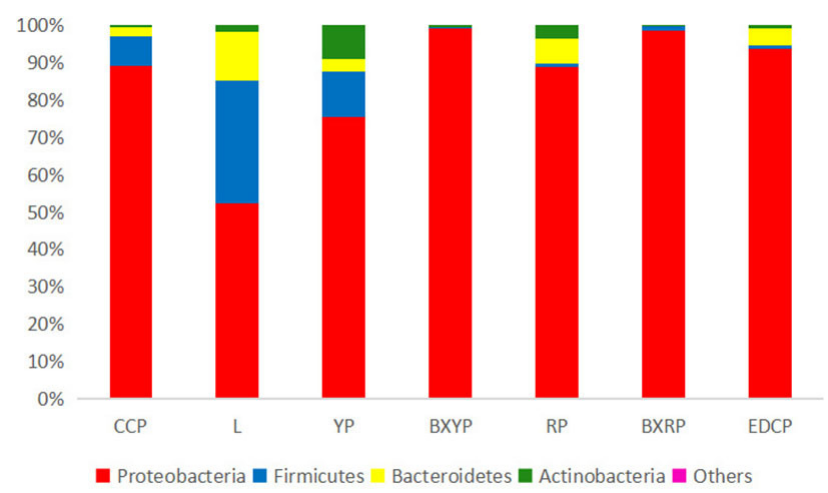
Microbial community bar plot of bacterial phylum of seven samples (for the best view, relative abundances of <0.05% are merged into other).

A total of 22 bacterial phyla were identified in *D. nuttalli*, with 9 phyla found in 7 growth stages, only 4 phyla (Proteobacteria, Firmicutes, Bacteroidetes, and Actinobacteria) with an abundance of >1% in every growth stage. Proteobacteria had the highest relative abundance, with a total abundance of 85.10% in all samples and relative abundances of 52.27–98.93% in different growth stages, indicating a marked predominance. Firmicutes had a total abundance of 8.12% in all samples and the highest relative abundances in eggs (32.76%) but only 0.33% in engorged larval ticks. In addition, the total abundances of Bacteroidetes and Actinobacteria in all samples were 4.31 and 2.43%, respectively, and the relative abundances of these families in different growth stages were 0.04–13.03% and 0.21–9.04%, respectively (Figure 4; Table 4).

**Table 4:** The relative abundance of bacterial phylum in the 7 samples.

**Bacterial phylum**	**Relative abundance (%)**							
	**Female adult**	**Eggs**	**Larval**	**Engorged**	**Nymphal**	**Engorged**	**Second-generation**	**Total**
	**ticks**		**ticks**	**larval ticks**	**ticks**	**nymphal ticks**	**adult ticks**	**abundance**
Proteobacteria	88.92	52.27	75.17	98.93	88.59	98.34	93.47	85.1
Firmicutes	7.85	32.76	12.41	0.33	1.14	1.37	0.97	8.12
Bacteroidetes	2.45	13.03	3.36	0.07	6.66	0.04	4.53	4.31
Actinobacteria	0.77	1.85	9.04	0.64	3.48	0.21	1.02	2.43
Other	0.01	0.08	0.03	0.03	0.13	0.04	0.02	0.05

#### Microbial community composition at the genus level

**Figure 5:**
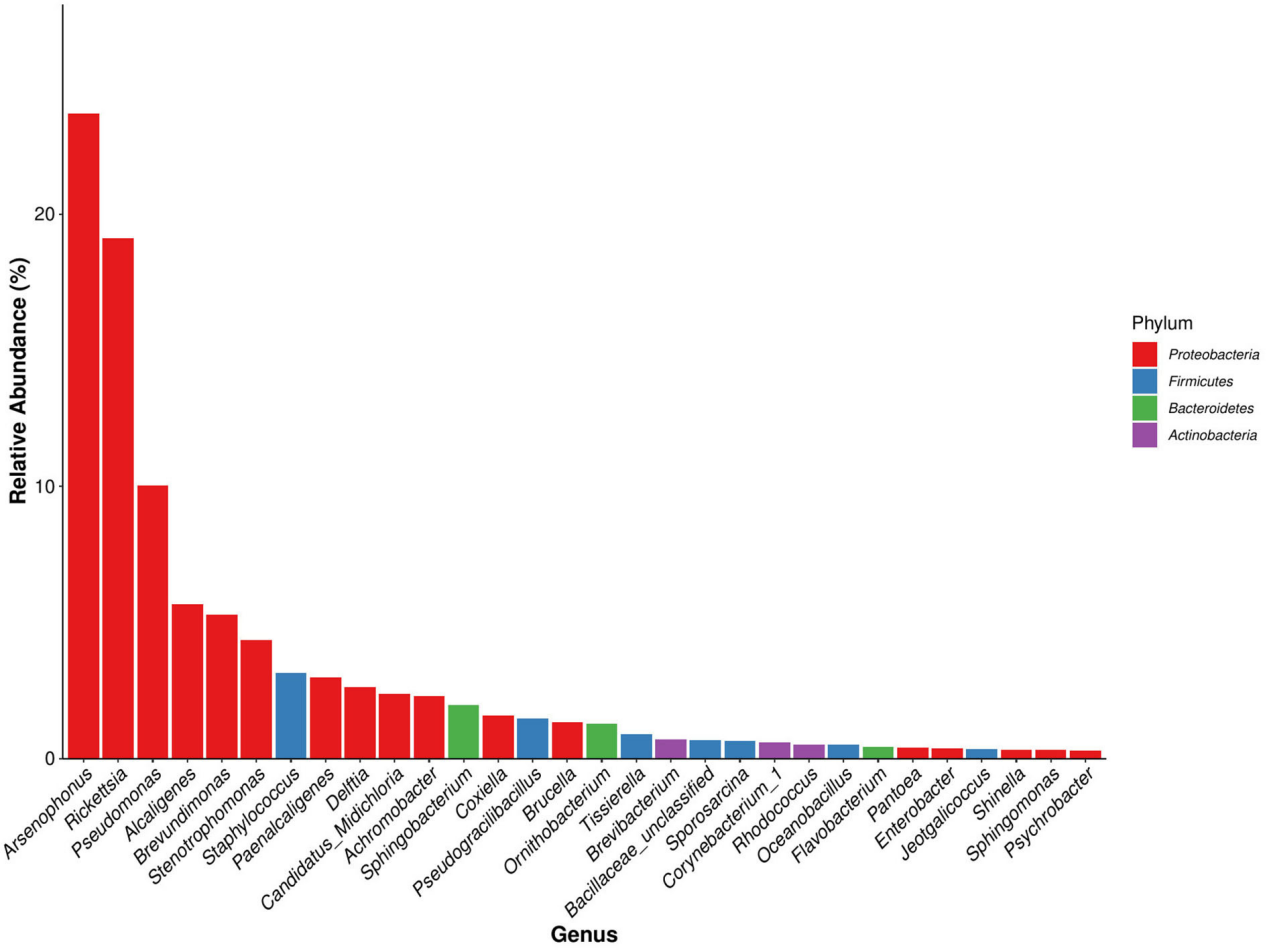
Dominant species bar plot of bacterial genus of *Dermacentor nuttalli* (a bacterial genus of the same color indicates that it is derived from the same bacterial phylum).

A total of 387 bacterial genera were identified in *D. nuttalli*, and 57 genera were present in every growth stage of *D. nuttalli*. There were 30 bacterial genera with high relative abundances in all samples of *D. nuttalli* (16 genera with relative abundances of >1%), 17 of which belonged to Proteobacteria. The top six bacterial genus in terms of relative abundance belonged to Proteobacteria (Figure 5). The bacterial genus with the highest relative abundance was *Arsenophonus*, with a total abundance of 23.70% in all samples. There were large differences in abundances in different growth stages as well as a high abundance in engorged ticks and larval ticks, wherein the relative abundances were 64.94 and 54.90%, respectively, and the lowest relative abundance was found in female adult ticks (0.15%). *Rickettsia* was the second most abundant bacterial genus in *D. nuttalli* (total abundance of 19.12%) and was found in all growth stages, with the highest relative abundance being the engorged larva stage (38.79%), followed by the engorged nymph stage (29.06%), and lower relative abundances in eggs and nymphal ticks (3.04 and 4.69%, respectively; the abundances in both adult stages were approximately the same (23.22% for female adult ticks and 23.38% for second-generation adult ticks). *Pseudomonas* was found primarily in female adult ticks (31.30%) and nymphal ticks (27.76%), with low relative abundances in engorged larval ticks (0.06%) and engorged nymphal ticks (0.01%). *Alcaligenes* had the highest relative abundance in second-generation adult ticks (17.36%), but abundance was only 0.005% in female adult ticks. *Brevundimonas* had the highest relative abundance in nymphal ticks (14.99%) but disappeared completely in the engorged nymph stage (0.008%). *Stenotrophomonas* was mainly found in female adult ticks (15.28%) and in low abundance in engorged larval ticks and engorged nymphal ticks (0.01 and 0.003%, respectively). Some common zoonotic pathogenic bacterial genus such as *Staphylococcus, Coxiella*, and *Brucella* were also detected, with total abundances of 3.14, 1.58, and 1.32% in all samples of *D. nuttalli*, respectively (Table 5).

**Table 5:** The relative abundance of 30 bacterial genera in the 7 samples.

**Bacterial genus**	**Relative abundance (%)**							
	**Female adult**	**Eggs**	**Larval**	**Engorged**	**Nymphal**	**Engorged**	**Second-generation**	**Total**
	**ticks**		**ticks**	**larval ticks**	**ticks**	**nymphal ticks**	**adult ticks**	**abundance**
*Arsenophonus*	0.151	4.458	38.050	54.904	1.360	64.937	2.008	23.695
*Rickettsia*	23.217	3.038	11.642	38.790	4.689	29.065	23.376	19.117
*Pseudomonas*	31.297	1.297	1.783	0.062	27.762	0.011	8.071	10.040
*Alcaligenes*	0.005	5.530	6.056	2.488	8.179	0.015	17.365	5.662
*Brevundimonas*	4.910	2.610	4.727	0.159	14.991	0.008	9.633	5.291
*Stenotrophomonas*	15.284	–	1.146	0.013	8.268	0.003	5.774	4.355
*Staphylococcus*	7.722	1.735	9.650	0.175	0.731	1.210	0.752	3.139
*Paenalcaligenes*	–	20.630	–	–	0.273	–	–	2.986
*Delftia*	0.047	0.002	0.139	0.017	11.285	0.008	6.945	2.635
*Candidatus Midichloria*	7.305	0.512	0.616	0.758	0.060	3.791	3.690	2.390
*Achromobacter*	0.522	0.002	1.278	0.024	5.168	0.000	9.010	2.286
*Sphingobacterium*	2.415	0.391	3.071	0.036	4.423	0.003	3.471	1.973
*Coxiella*	1.518	8.011	1.068	0.233	0.045	0.050	0.151	1.583
*Pseudogracilibacillus*	–	10.055	0.112	0.000	0.107	–	–	1.468
*Brucella*	2.630	0.950	2.261	0.095	1.791	0.050	1.477	1.322
*Ornithobacterium*	–	9.030	–	–	–	–	–	1.290
*Tissierella*	–	6.307	0.018	–	0.004	–	–	0.904
*Brevibacterium*	0.387	0.180	2.143	0.059	1.572	0.005	0.587	0.705
*Bacillaceae unclassified*	–	4.655	0.024	0.001	0.016	–	–	0.671
*Sporosarcina*	–	4.533	0.001	0.004	0.052	–	–	0.656
*Corynebacterium* 1	0.100	0.941	2.346	0.130	0.577	0.012	0.054	0.594
*Rhodococcus*	0.174	0.042	2.335	0.308	0.507	0.142	0.107	0.516
*Oceanobacillus*	–	3.522	0.001	0.000	0.004	–	–	0.504
*Flavobacterium*	0.001	–	0.009	–	1.966	0.000	1.003	0.426
*Pantoea*	0.012	0.000	2.687	0.048	0.008	0.000	–	0.394
*Enterobacter*	0.076	0.001	1.404	0.174	0.360	0.134	0.454	0.372
*Jeotgalicoccus*	0.017	0.187	2.077	0.016	0.045	0.037	0.038	0.345
*Shinella*	0.213	0.006	0.226	0.004	1.000	0.007	0.836	0.328
*Sphingomonas*	0.003	0.007	0.038	0.018	0.523	0.062	1.571	0.317
*Psychrobacter*	0.543	0.700	0.796	0.003	0.000	–	–	0.292

“—” indicates that the bacterial genus was not detected in any of the three replicate samples at each growth stage of D. nuttalli; “0.000” indicates that the bacterial genus was detected, but its abundance was extremely low; “Unclassified” indicates that the database could not be compared with the database under the confidence threshold.

Some bacterial genera were present in specific growth stages, e.g., *Stenotrophomonas* was found in all six growth stages but not in eggs; *Paenalcaligenes* was detected in eggs and nymphal ticks but not in other stages; *Pseudogracilibacillus* was detected in eggs, larval ticks, engorged larval ticks, and nymphal ticks but not in female adult ticks, engorged nymphal ticks, and second-generation adult ticks; and *Ornithobacterium* was found only in eggs. Some bacterial genera with total abundances of <1% were also present in some growth stages, including *Tissierella, Bacillaceae* unclassified, *Sporosarcina, Oceanobacillus, Flavobacterium, Pantoea*, and *Psychrobacter* (Table 5).

**Figure 6:**
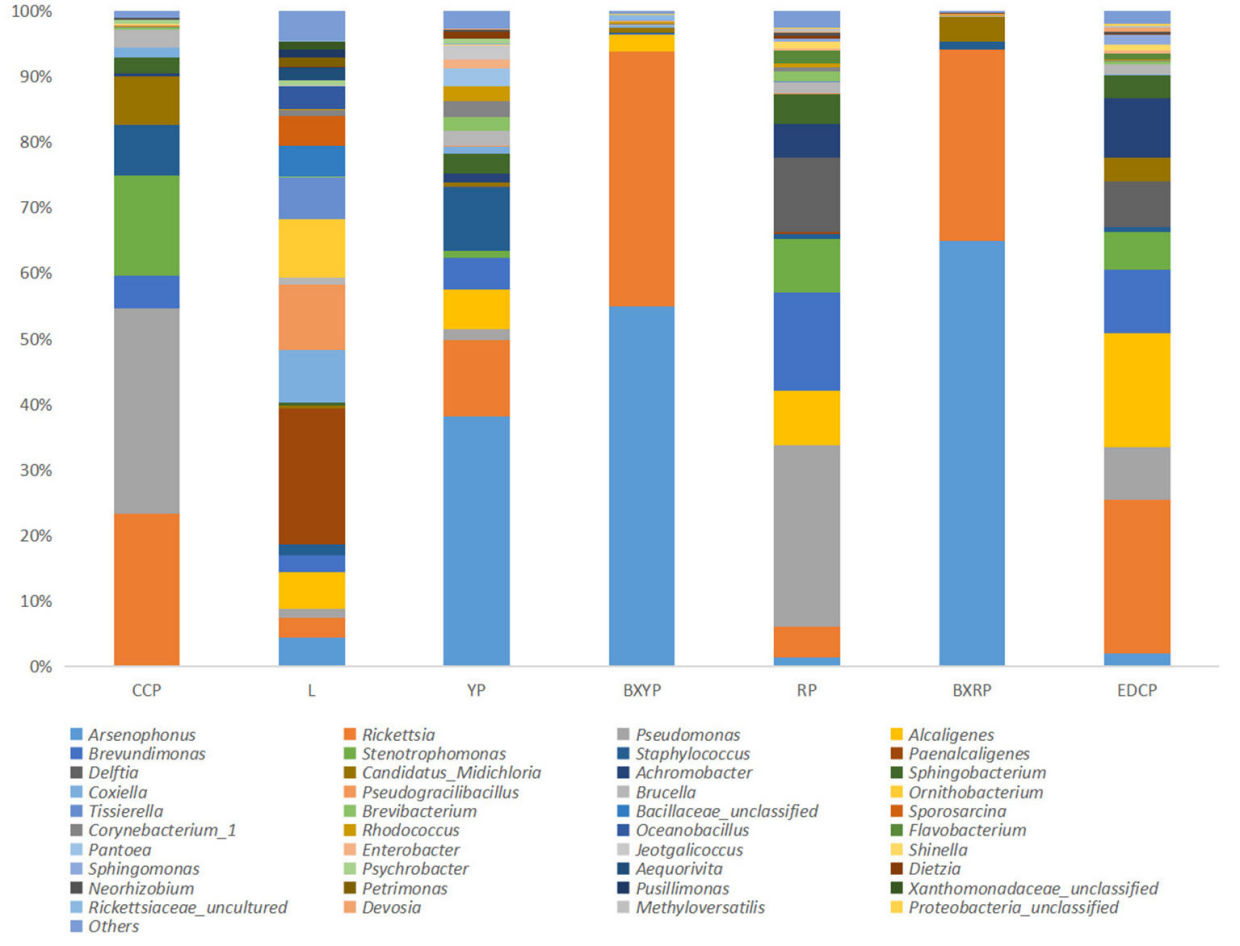
Microbial community bar plot of bacterial genus of seven samples.

The dominant genera of *D. nuttalli* at different growth stages differed from the overall dominant genera of all samples of *D. nuttalli*. The genera with higher relative abundances among female adult ticks were *Pseudomonas* (31.30%), *Rickettsia* (23.22%), *Stenotrophomonas* (15.28%), *Staphylococcus* (7.72%), and *Candidatus Midichloria* (7.31%). The genera with the highest relative abundances of eggs were *Paenalcaligenes* (20.63%), *Pseudogracilibacillus* (10.06%), *Ornithobacterium* (9.03%), and *Coxiella* (8.01%). *Arsenophonus* had the highest relative abundances of both larval ticks and engorged larval ticks (38.05 and 54.90%, respectively). The relative abundance of *Rickettsia* increased from 11.64% in the larval tick stage to 38.79% in the engorged larval tick stage. The relative abundance of *Staphylococcus* decreased from 9.65% in the larval tick stage to 0.18% in the engorged larval tick stage. *Pseudomonas* (27.76%), *Brevundimonas* (14.99%), *Delftia* (11.29%), and *Stenotrophomonas* (8.27%) were the genera with higher relative abundances among the nymphal ticks. The dominant genera in the engorged nymph stage were *Arsenophonus* (64.94%) and *Rickettsia* (29.07%). In the second generation of adult ticks, the genus with the highest relative abundance was *Rickettsia* (23.38%), *Alcaligenes* (17.37%), *Brevundimonas* (9.63%), *Achromobacter* (9.01%), and *Pseudomonas* (8.07%) (Figure 6).

#### Microbial community composition at the species level

*Dermacentor nuttalli* was associated with 23 bacterial species, with *Alcaligenes faecalis, Brevundimonas diminuta, Delftia tsuruhatensis, Achromobacter xylosoxidans* subsp. *xylosoxidans, Staphylococcus xylosus, Brucella melitensis, Sphingobacterium mizutaii, Staphylococcus sciuri* subsp. *sciuri* accounting for more than 1% of the total. Total abundances were higher in *Alcaligenes faecalis* and *Brevundimonas diminuta*, where the abundances in the larval tick and nymphal tick stages were significantly higher in their engorged stages and were also significantly higher in the second generation of adult ticks. The abundances of *Delftia tsuruhatensis* and *Achromobacter xylosoxidans* subsp. *xylosoxidans* were higher in the nymph and second-generation adult tick stages. *Staphylococcus xylosus* and *Brucella melitensis* were more abundant in female adult and larval tick stages than in any other stages (Table 6).

**Table 6:** The relative abundance of 23 bacterial species in the 7 samples.

**Bacterial species**	**Relative abundance (%)**							
	**Female adult**	**Eggs**	**Larval**	**Engorged**	**Nymphal**	**Engorged**	**Second-generation**	**Total**
	**ticks**		**ticks**	**larval ticks**	**ticks**	**nymphal ticks**	**adult ticks**	**abundance**
*Alcaligenes faecalis*	0.005	5.530	6.055	2.488	8.171	0.015	17.218	5.640
*Brevundimonas diminuta*	4.583	2.185	3.874	0.152	14.001	0.005	8.805	4.801
*Delftia tsuruhatensis*	0.047	0.002	0.139	0.017	11.116	0.008	6.941	2.610
*Achromobacter xylosoxidans* subsp. *xylosoxidans*	0.522	0.002	1.278	0.024	5.168	0.000	9.010	2.286
*Staphylococcus xylosus*	6.997	1.203	5.158	0.090	0.342	0.978	0.170	2.134
*Brucella melitensis*	2.630	0.950	2.261	0.095	1.791	0.050	1.477	1.322
*Sphingobacterium mizutaii*	2.152	0.005	1.428	0.017	2.548	0.003	1.140	1.042
*Staphylococcus sciuri* subsp. *sciuri*	0.725	0.532	4.491	0.086	0.389	0.233	0.583	1.005
*Brevibacterium epidermidis*	0.359	0.156	2.129	0.059	1.565	0.005	0.584	0.694
*Corynebacterium stationis*	0.097	0.939	2.275	0.123	0.571	0.000	0.050	0.579
*Rhodococcus erythropolis*	0.174	0.042	2.327	0.307	0.495	0.142	0.107	0.513
*Brachybacterium paraconglomeratum*	0.065	0.116	0.369	0.018	0.119	0.001	0.149	0.120
*Microbacterium oxydans*	0.021	0.002	0.075	0.002	0.099	0.004	0.023	0.032
*Sphingomonas panni*	0.001	0.007	0.025	0.015	0.033	0.062	0.031	0.025
*Methylobacterium populi*	0.001	0.000	0.008	0.002	0.011	0.011	0.045	0.011
*Lactobacillus johnsonii*	0.003	0.006	0.004	0.003	0.009	0.003	0.009	0.005
*Halomonas stevensii S18214*	0.001	0.000	0.007	0.003	0.001	0.016	0.009	0.005
*Lactobacillus murinus*	0.003	0.001	0.006	0.007	0.004	0.004	0.004	0.004
*Arthrobacter* sp. *IHBB 11108*	0.000	0.000	0.011	0.002	0.003	0.007	0.003	0.004
*Ruminococcus* sp. *5 1 39BFAA*	0.003	0.003	0.004	0.005	0.002	0.003	0.004	0.003
*Lactobacillus reuteri*	0.003	0.002	0.001	0.005	0.003	0.005	0.003	0.003
*Streptococcus salivarius* subsp. *thermophilus*	0.010	0.000	0.002	0.002	0.000	0.002	0.003	0.003
*Bifidobacterium animalis*	0.001	0.002	0.001	0.005	0.003	0.001	0.001	0.002

“0.000” indicates that the strain was detected but in very low abundance.

## Discussion

China reported 124 tick species, 103 tick-borne agents, and 29 tick-borne pathogens infecting humans from 1950 to 2018 (3). In the 33 years since 1982, Mainland China has identified 33 emerging tick-associated agents, of which 20 have been identified to cause human diseases (10). Different tick species prefer different biotopes or environments, which influences their geographical distribution and, as a result, the risk area for human tick-borne infections. *D. nuttalli* is ubiquitous in northern China (11, 50), one of the dominant tick species in Inner Mongolia and is one of the most dangerous tick species in Inner Mongolia in terms of public health (11). It spreads multiple pathogens and causes multiple infectious diseases (10, 34). Current research on the carriage and transmission of pathogens by *D. nuttalli* focuses primarily on the detection of one specific pathogen carried by *D. nuttalli* adult ticks (15–33) and different developmental stages of *D. nuttalli* (34–40); however, a few studies have employed next-generation sequencing on *D. nuttalli* adult ticks (41–44). The 16S rDNA amplicon sequencing has advantages, such as the identification of low-abundance and non-culturable bacteria (44). The microbial community structure and diversity of different growth stages of *D. nuttalli* have not yet been comprehensively studied. As the Hulunbuir League is one of the most important pastoral regions in Inner Mongolia, hard ticks thrive there (41). In this study, we investigated the microbial community structures of *D. nuttalli* at seven growth stages in the Hulunbuir League region of China using high-throughput sequencing.

In the alpha diversity of community ecology, a higher diversity in the sample microbial community was indicated by higher Shannon Index and lower Simpson Index values. Higher ACE values indicate a greater total number of species (51). The sequencing results showed that the egg stage had the greatest diversity and the highest number of species, while the engorged nymph stage had the least diversity and number of species. The sequencing depth index (Good's coverage) of all 7 growth stage samples exceeded 99.9%, indicating that the sequencing depth is sufficient to demonstrate the microbial diversity of the samples and can reflect the actual situation of microorganisms carried by the samples. The species accumulation curves also reflected the abundance of the *D. nuttalli* microbial community as the sample size required to detect a large number of species in the community. OTU cluster analysis showed that some microbial populations were identical among the seven growth stages of *D. nuttalli*. On the basis of the above estimators, analysis of the sequencing data from this study showed that 387 genera of 22 phyla were associated with *D. nuttalli*, of which 57 genera of 9 phyla were found in all 7 growth stages. Proteobacteria are the dominant phylum of *D. nuttalli* and are consistent with the dominant phylum of *Haemaphysalis flava* (13), *Amblyomma maculatum* (52), *Amblyomma tuberculatum* (53), *Haemaphysalis longicornis* (54), and *D. nuttalli* in Guertu County, Wusu City (44), which may be related to the widespread distribution of Proteobacteria in nature and suggests that Proteobacteria are viable and abundant in different tick species. At the level of bacterial genera, the dominant genera of *D. nuttalli* in this study were *Arsenophonus* and *Rickettsia*. It has also been reported that *Rickettsia* was detected in *Haemaphysalis flava* (Xinyang city, Henan Province, China), *Amblyomma americanum* (Edwards and Sutton Counties, USA), *Rhipicephalus turanicus* (Kibbutz Hulda, Israel) and *Rhipicephalus sanguineus* (Caesarea) (13). This is not consistent with the dominant genera (*Pseudomonas* and *Coxiella*) reported for *D. nuttalli* in Guertu County (44) and may suggest regional differences.

In this study, *Arsenophonus* has the highest total abundance in *D. nuttalli* and the lowest abundance in the first-generation female adult tick stage (0.15%); its abundance increased from 4.46% for eggs and 38.05% for larval ticks to 54.90% for engorged larval ticks but then dropped to 1.36% in the nymph stage and increased again to a peak of 64.94% in engorged nymphal ticks before decreasing to 2.01% in the second generation of adult ticks. According to the literature, blood-feeding behavior alters the composition and abundance of the microbiome in insects (12). Studies have shown that the community diversity of *Anopheles gambiae* significantly decreased (55) and the midgut microbiota of *Aedes aegypti* increased after blood meals (56); blood feeding reduces the microbial richness of the sand fly midgut and stimulates the proliferation (57, 58) and diversification of colonizing female midge intestinal bacterial populations (59); and blood sucking increases the relative abundance of *Bartonella* while decreasing *Wolbachia* and most other bacterial genera after bloodsucking *Melophagus ovinus* (51). According to the literature, the process of imbibing blood appears to affect the tick microbiome (60), and blood meal feeding usually reduces the diversity of internal tick microbiota (44). The results of this study showed that the act of blood sucking significantly increased the relative abundance of *Arsenophonus*. *Arsenophonus* is an intracellular symbiotic bacterium that infects a variety of arthropods and plays an important role in killing male host insects while also providing vitamins and other nutrients to host insects (61). The role of *Arsenophonus* in ticks is not well understood (62). Further studies are needed to determine the effect of bloodsucking behavior on *Arsenophonus in D. nuttalli* and the role of *Arsenophonus* in *D. nuttalli*.

In this study, *Rickettsia* was the second most abundant bacterial genus of *D. nuttalli* in terms of total abundance. *Rickettsia* is an obligate, intracellular, parasitic bacterium that is spread mainly by arthropods (61). *Rickettsia* is also considered a symbiont in the broad sense and has been found in high concentrations in a number of ticks, including *Rhipicephalus microplus* (63), *Ixodes scapularis* (64), *Rhipicephalus turanicus*, and *Rhipicephalus sanguineus*. In previous studies, spotted fever group *Rickettsiae* were detected in up to 67.4% of *D. nuttalli* and potential novel *Rickettsia* species were found (65). Furthermore, *Rickettsia raoultii* may be the predominant *Rickettsia* in *D. nuttalli*. Additionally, *Rickettsia raoultii* and *Rickettsia sibirica* subsp. *Sibirica* causes Siberian tick typhus, which has been found in nymphal ticks as well as in both unfed and engorged adults of *D. nuttalli* (23, 27, 39, 66). The pathogen of Q fever was also detected in unfed *D. nuttalli* ticks and in *D. nuttalli* ticks collected from livestock (26). *Rickettsia sibirica, Rickettsia slovaca* (23), *Rickettsia* sp. 10CYF, *Candidatus Rickettsia tibetani* (27), *Rickettsia tarasevichiae* (41), *Rickettsia aeschlimannii* (18), *Rickettsia heilongjiangensis* (10), and others were also detected in *D. nuttalli*. The above study showed that *D. nuttalli* carried a high abundance and a variety of *Rickettsia*. The results of this study showed an increased abundance of *Rickettsia* after a blood meal, which is consistent with the abundance of *Rickettsia* in unfed ticks (*D. nuttalli*) in Guertu County being <5% (44). The difference in *Rickettsia* abundance in *D. nuttalli* in different reports may also be related to the geographical location of the samples. The ability of another arthropod, *M. ovinus* carrying *Rickettsia*, also reflects geographic regional differences (61). Many *Rickettsia* are tick-borne pathogens that cause tick-borne rickettsial diseases, which pose a serious threat to human and animal health worldwide. However, in ticks, these *Rickettsia* are not pathogenic and exist only as maternally inherited symbionts with an intimate (but not necessarily beneficial) relationship with the host (67), which is consistent with the present study, in which *Rickettsia* were found in all seven different growth stages. It has also been reported that *Rickettsia* switching between pathogenic and non-pathogenic forms and between hosts is a common feature of *Rickettsia* evolution, which may lead to the emergence of new infectious diseases (68). In summary, arthropod *Rickettsia* may require more of our attention.

Additionally, in the present study, there were many other bacterial genera that were presented in various stages, including eggs, indicating that these microorganisms may be transovarially transmitted and may be in a symbiotic relationship with *D. nuttalli*, despite the fact that these bacteria do not appear to be pathogenic in ticks, as reported in the literature (69). Similarly, the pathogenicity of these microorganisms to humans and animals cannot be overlooked. After engorgement in larval ticks and nymphal ticks, the abundance of *Pseudomonas, Alcaligenes, Brevundimonas*, and *Stenotrophomonas* decreased to varying degrees. This coincides with the highest abundance of *Pseudomonas* in the unfed ticks *(D. nuttalli*) in Guertu County (44). It has been previously reported that sheep sera appears to lyse *Borrelia garinii* and *Borrelia valaisiana* (70), but the relationship of blood to the decreased abundance of these microorganisms in *D. nuttalli* is unknown. In this study, a large number of microorganisms were annotated in different growth stages of *D. nuttalli*, but the dominant genera in each growth stage were different, e.g., the dominant genus in eggs was *Paenalcaligenes*, but only a low abundance of *Paenalcaligenes* was found in the nymph stage, whereas the abundance of *Paenalcaligenes* in houseflies increased with increased larval development (71). All of this evidence suggests that the microbial community composition and diversity in ticks are dynamic and are influenced by a variety of factors, including tick developmental stage (72), geographical location (73), blood sucking (74), engorgement status (75, 76), sex (77), host, and tick species (12, 78). In conclusion, the microbial community structure of *D. nuttalli* exhibits differential and stage-specific characteristics.

Finally, we would like to discuss a few species with higher abundance in the 23 specific bacterial species that were only annotated to *D. nuttalli*. *Alcaligenes faecalis* is an obligate aerobic, gram-negative rod that causes skin and soft tissue infections, meningitis, endocarditis, chronic otitis, bacteremia, pyelonephritis, endophthalmitis, peritonitis, abscesses (79), urinary tract infections, and pneumonia (80). Most infections caused by this organism are nosocomial, occur in immunocompromised hosts, and have a high level of resistance to commonly used antibiotics (79). *Brevundimonas diminuta* is a gram-negative aerobic bacillus. It causes bacteremia, urinary tract infection, pleuritis, empyema, peritonitis, keratitis, bloodstream infection, nephrotic syndrome, post-traumatic abscess, Leg ulcer (81), and pyogenic liver abscesses and is typically resistant to a wide range of antimicrobials (82). *Delftia tsuruhatensis* is a gram-negative bacillus discovered in Japan in 2003. These bacteria cause bacteremia (83), catheter-related infection (84), respiratory infection in a premature infant (85), and are resistant to a variety of antibiotics (83, 84). *Achromobacter xylosoxidans*, formerly known as *Alcaligenes denitrificans* subsp. *xylosoxidans*, is a gram-negative bacillus that is intrinsically resistant to most antibiotics and was first found in the purulent ear drainage of patients with chronic otitis media. It was later identified as an etiologic agent of meningitis, pneumonia, surgical wound infections, bacteremia, septicemia, urinary tract infections, peritonitis, pharyngitis, etiologic agent (86), and endocarditis (87). Patients with cystic fibrosis and patients with non-cystic fibrosis (patients with bronchiectasis and patients with cancer) are at risk of infection with *Achromobacter xylosoxidans* (88), and there is evidence of patient-to-patient transmission (87). *Brucella melitensis* is a major pathogen of brucellosis in goats and sheep and is the most common pathogen in human brucellosis (89). In animals, *Brucella* infection has a negative impact on fetal development and reproductive organs, leading to reproductive failure, abortions, and infertility (90). The Food and Agriculture Organization of the United Nations, the World Health Organization, and the Office International des Epizootics consider brucellosis to be one of the world's most serious neglected zoonotic diseases (90). Ticks are reservoir vectors for brucellosis and genes specific to the *Brucella* genus have been found in developmental stages and anatomical regions of ticks. *B. melitensis* was isolated from eggs and engorged adults of *D. nuttalli*. *Dermacentor nuttalli* is a potent, long-term carrier of *Brucella* spp. and shows transovarial transmission potential (34), indicating the potential risk of transmission of brucellosis between animals and humans *via* tick bites. *Staphylococcus xylosus* is a commensal gram-positive bacterium found on human and animal skin and is a clinically relevant pathogen in veterinary medicine (91). *Sphingobacterium mizutaii* is a gram-negative organism that is very similar to *Sphingobacterium mizutaii*, which has been isolated from patients with cellulitis (92). *Staphylococcus sciuri* is thought to be a commensal animal-associated species, but it has been isolated from bovine mastitis, goats with ovine rinderpest, canine dermatitis (93), and piglets with fatal exudative epidermitis. These bacteria cause lesions of the lungs and endocarditis in piglets; are fatal to mice and newly born piglets (94); may be related to human endocarditis, endophthalmitis, skin wound infection, peritonitis, pelvic inflammatory disease, septic shock, urinary tract infections, and wound infections; and are multidrug-resistant bacteria (95). These microorganisms that we identified in *D. nuttalli* deserve further investigation, and it is necessary to conduct surveillance of some highly abundant bacteria in *D. nuttalli* that require close monitoring because of their potential to cause disease in humans or animals.

## Conclusions

The egg stage of *D. nuttalli* in Inner Mongolia, China, had the most diversity and species, with 387 genera and 23 species of 22 phyla, of which 9 phyla, 57 genera, and 23 species were found in all 7 growth stages of *D. nuttalli*. The dominant phylum was Proteobacteria, and the dominant bacterial genera were *Arsenophonus* and *Rickettsia*. The dominant genera differ at different growth stages. In this study, the microbial community composition of different growth stages of *D. nuttalli* was comprehensively analyzed for the first time.

## Data availability statement

The raw tags have been deposited in Sequence Read Archive (SRA) from the NCBI under BioProject accession number PRJNA848250. The individual run files received the accession numbers SRR19668431 ~ SRR19668451.

## Ethics statement

The Biomedical Research Ethics Committee of Inner Mongolia Agricultural University specifically approved this study [No. 2020(081)]. Written informed consent was obtained from the owners for the participation of their animals in this study.

## Author contributions

LZ, Y-MM, and Y-HL conceived and designed the study and critically revised the manuscript. BY, W-XH, LZ, and Y-MM performed the sample collection. LZ, Y-MM, W-HZ, H-LC, Z-SZ, Y-JZ, J-LW, Y-LD, and Y-HL conducted the laboratory experiments. Y-HL, LZ, Y-MM, YX, and L-FY conducted sequencing and participated in sequence analysis. All authors read and approved the final manuscript.

## Funding

This study was funded by the Inner Mongolia Agricultural University High-level Talents Research Initiation Fund Project (NDYB2019-3 and NDYB2018-5), National Natural Science Foundation of China (32260887 and 31860698), National Natural Science Foundation of Inner Mongolia (2022MS03023), and supported by State Key Laboratory of Veterinary Biotechnology Foundation (SKLVBF202204).

## Conflict of interest

Author W-XH was employed by Inner Mongolia Saikexing Reproductive Biotechnology (Group) Co., Ltd., Hohhot, China. Authors YX and L-FY were employed by Shanghai Origingene Bio-pharm Technology Co. Ltd., Shanghai, China. The remaining authors declare that the research was conducted in the absence of any commercial or financial relationships that could be construed as a potential conflict of interest.

## Publisher's note

All claims expressed in this article are solely those of the authors and do not necessarily represent those of their affiliated organizations, or those of the publisher, the editors and the reviewers. Any product that may be evaluated in this article, or claim that may be made by its manufacturer, is not guaranteed or endorsed by the publisher.
